# Nasal double DNA adjuvant induces salivary FimA-specific secretory IgA antibodies in young and aging mice and blocks *Porphyromonas gingivalis* binding to a salivary protein

**DOI:** 10.1186/s12903-019-0886-2

**Published:** 2019-08-19

**Authors:** Kenjiro Kobuchi, Kosuke Kataoka, Yoichiro Taguchi, Tatsuro Miyake, Makoto Umeda

**Affiliations:** 1 Graduate School of Dentistry, Osaka Dental University, Hirakata, Osaka, 573-1121 Japan; 2 Department of Preventive and Community Dentistry, Osaka Dental University, Hirakata, Osaka, 573-1121 Japan; 3 Department of Periodontology, Osaka Dental University, Hirakata, Osaka, 573-1121 Japan

**Keywords:** CpG ODN, Dendritic cells, DNA plasmid, FimA, FL gene, Nasal adjuvant, Nasal immunization, Salivary SIgA

## Abstract

**Background:**

We previously showed that nasal administration of a combination of dendritic cell (DC) targeted DNA plasmid expressing Flt3 ligand and CpG oligodeoxynucleotides 1826 as a mucosal adjuvant (double adjuvant, DA) provoked protective immunity in the upper respiratory tract of young adult and aging mice. Here, we investigated whether the nasal DA system induces secretory (S)IgA antibodies (Abs) toward recombinant fimbrillin (*r*FimA) of *Porphyromonas gingivalis* (*P. gingivalis*) in the saliva of young adult and aging mice. Further, we examined the functional applicability of *r*FimA-specific salivary SIgA Abs*.*

**Methods:**

BALB/c mice (8- or 48-week-old) were nasally immunized with *r*FimA plus DA three times at weekly intervals. Control mice were nasally administered *r*FimA alone. Saliva samples were collected 1 week after the final immunization, and were subjected to *r*FimA-specific ELISA. To examine the functional applicability of *r*FimA-specific SIgA Abs, IgA-enriched saliva samples were subjected to an inhibition assay in order to assess the numbers of *P. gingivalis* cells bound to the salivary protein statherin.

**Results:**

The 8- and 48-week-old mice administered nasal *r*FimA plus DA showed significantly increased levels of *r*FimA-specific SIgA Abs in saliva and elevated numbers of CD11c^+^ DCs in sublingual glands (SLGs), periglandular lymph nodes (PGLNs) and submandibular glands (SMGs) as well as nasopharyngeal-associated lymphoid tissues (NALT) compared to mice administered *r*FimA alone. Further, *r*FimA-specific SIgA Abs-containing saliva, in which IgG Abs of 8- and 48-week-old mice administered nasal *r*FimA plus DA were removed, significantly inhibited binding of *P. gingivalis* to the salivary protein.

**Conclusions:**

These findings show that this DA system could be an effective nasal vaccine strategy for the enhancement of *P. gingivalis*-specific protective immunity in the oral cavity of adolescents and older individuals.

**Electronic supplementary material:**

The online version of this article (10.1186/s12903-019-0886-2) contains supplementary material, which is available to authorized users.

## Background

Periodontitis is one of the most prevalent infectious diseases worldwide and is characterized by the destruction of periodontal supportive tissues, including inflammation of the gingiva and alveolar bone loss, following periodontal-pathogenic bacterial infection and disturbance of host immunity. *Porphyromonas gingivalis* (*P. gingivalis*) is a Gram-negative anaerobic bacterium that forms black-pigmented colonies and is involved in the onset and progression of periodontal disease [1, 2] and multiple systemic diseases [3, 4], despite being detected in healthy people [5]. Fimbriae located on the cell surface of *P. gingivalis* are primarily composed of polymers of FimA protein (fimbrillin), encoded by the gene *fimA* [6]. It is known as a virulence factor [7], and plays an important role in colonization through its association with host tissues, including salivary proteins, and other bacteria in the oral cavity [8–10]. Moreover, it has been shown that fimA-inactivated mutants showed reduced abilities to adhere to human gingival fibroblasts and epithelial cells [11]. Recently, it has also been reported that the FimA protein elicited inflammatory responses via the TLR4/NF-*k*B signaling pathway in human peripheral blood mononuclear cells [12]. We also previously showed that *P. gingivalis* FimA protein binds specifically and rigidly to salivary statherin, a human salivary protein, in a solid phase system [13].

Nasal immunization can effectively evoke the nasopharyngeal-associated lymphoid tissues (NALT)-based immune system, which preferentially induces antigen (Ag)-specific antibody (Ab) responses in the oral cavity [14] and other mucosal tissues [15, 16]. A previous study showed that the NALT-based immune system of one-year-old mice exhibited intact mucosal immune responses when mice were administered cholera toxin as a nasal adjuvant, in contrast to the age-associate alterations in intestinal immune responses [17]. Although nasal cholera toxin is a potent mucosal adjuvant, the development of safer nasal adjuvants to induce protective Ag-specific immune responses in mucosal compartments, including the oral cavity, is needed [18, 19]. In this regard, we have shown that nasal application of a DNA plasmid encoding Flt3 ligand cDNA (pFL) preferentially expanded CD8^+^ CD11c^+^ dendritic cells (DCs) and subsequently induced IL-4-producing CD4^+^ T cell-mediated Ag-specific mucosal immune responses [20, 21]. Further, we showed that a nasal double adjuvant (DA) composed of pFL and CpG oligodeoxynucleotides (CpG ODN) enhanced mucosal and systemic immune responses with increased numbers of CD8^+^ and B220^+^ CD11c^+^ DC subsets in the nasal mucosa [22, 23].

IgA Abs are produced as the major isotype on mucosal surfaces (approximately 200 mg/L of SIgA, 2 mg/L of IgG and 1 mg/L of IgM in human whole saliva) [24], which is mainly secreted as the dimeric or polymeric form of IgA (secretory IgA, SIgA) Abs. It has been shown that the major roles of SIgA Abs are neutralization of viruses or toxins and inhibition of bacterial adherence to host mucosal and tooth surfaces [24]. It has been shown that the levels of serum IgG Abs to *P. gingivalis* in adult and early-onset periodontitis patients were higher compared to healthy individuals, and these Ab responses exhibited protective roles in the disease process [25]. Further, specific IgA Abs directed to periodontal-pathogenic microorganisms in the gingival crevicular fluid (GCF) played a protective role in the onset of periodontal disease [26]. Conversely, it was reported that healthy individuals possessed higher levels of serum *P. gingivalis*-specific IgG Abs compared to periodontal disease patients [27]. Despite these studies, the protective roles of *P. gingivalis*-specific IgG and IgA Abs in periodontal disease remains unclear. In this study, we examined whether nasal DA could induce SIgA Abs to *r*Fim A of *P. gingivalis* in the saliva of young adult and aging mice. Further, we determined the ability of *r*FimA-specific SIgA Abs to block adherence of live *P. gingivalis* to a salivary protein*.* We consequently showed that nasal *r*FimA plus DA successfully elicits elevated levels of *r*FimA-specific salivary SIgA Ab responses and expands mucosal CD11c^+^ DCs in both young adult and aging mice. Further, IgG Ab-depleted saliva from young adult and aging mice given nasal *r*FimA plus DA showed significantly decreased binding of *P. gingivalis* to a statherin-coated plate compared to mice administered nasal *r*FimA alone.

## Methods

### Mice

Female BALB/c mice were obtained from SLC Japan, Hamamatsu, Japan. Five mice per cage were maintained in horizontal laminar flow cabinets and were provided sterile food and water as part of a specific pathogen-free facility at Osaka Dental University (Hirakata, Japan). All experiments were conducted in accordance with the guidelines provided by Osaka Dental University. All mice in this study were 8 or 48 weeks of age.

### Recombinant FimA (*r*FimA)

**Fig. 1:**
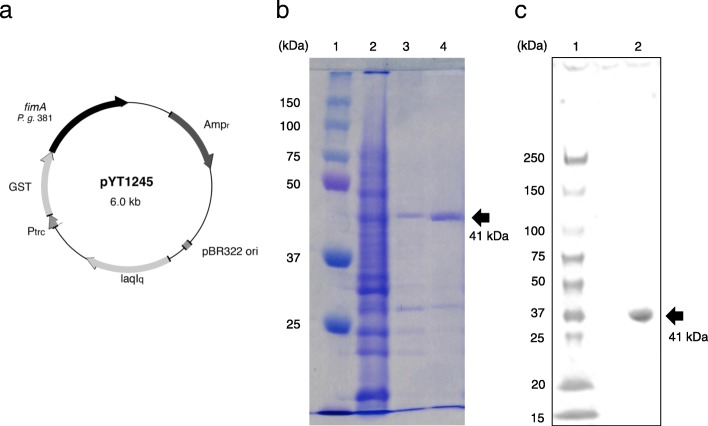
Expression and purification of *r*FimA. (**a**) *r*FimA expression was induced in *E. coli* BL21 competent cells by transformation with the DNA plasmid PYT1245 using the heat shock method. (**b**) Ampicillin-resistance transformants were induced by Isopropyl-β-D-thiogalactopyranoside (IPTG), and disrupted with ultrasonication. The supernatants from ultrasonicated *E. coli* BL21 containing Glutathione S-transferase (GST)-*r*FimA fusion proteins were applied to a GST affinity column. *r*FimA protein was eluted by cleaving the GST domain from *r*FimA using PreScission Protease. The purified *r*FimA protein was electrophoresed in a 4–20% gradient SDS-PAGE gel and stained with Coomassie brilliant blue R-250. Lane 1: standard marker, Lane 2: the supernatants from the ultrasonicated transformant, Lane 3: eluted solution (1 μg) from GST affinity column, Lane 4: eluted solution (3 μg) from the GST affinity column. (**c**) The purity of *r*FimA was confirmed by western blot analysis. *r*FimA (5 μg) was transferred to a PVDF membrane and detected using rabbit anti-fimbriae serum (1: 2000 dilution) followed by HRP-conjugated goat anti-rabbit IgG Ab and TMB substrate solution. Lane 1; protein molecular weight marker, Lane 2; purified *r*FimA

The DNA plasmid vector PYT1245 (Fig. 1a) expressing FimA and anti-fimbriae rabbit serum was kindly provided by Dr. Yutaka Terao of Niigata University [28, 29]. DNA manipulations were carried out according to the manufacturer’s instructions. *Escherichia coli* BL21 competent cells (BioDynamics Laboratory Inc., Tokyo, Japan) were transformed with PYT1245 by the heat-shocking method and were cultured in Luria-Bertani medium supplemented with ampicillin (100 μg/mL). The supernatants from ultrasonicated *E. coli* BL21 transformants carrying the PYT1245 plasmid (Fig. 1b, lane 2) were applied to a GST affinity column (GE Healthcare Bio-Sciences AB, Uppsala, Sweden). The *r*FimA protein (MW; approximately 41 kDa) was eluted by cleaving the GST-*r*FimA fusion protein with PreScission protease™ (GE Healthcare Bio-Sciences AB) (Fig. 1b, lane 3; 1 μg and lane 4; 3 μg). The recovered protein was employed as the purified *r*FImA protein in this study.

### *r*FimA-specific western blotting analysis

In order to confirm the purity of *r*FimA, western blotting analysis (Fig. 1c) was performed using rabbit anti-FimA serum as described previously [24]. In brief, the proteins were transferred electrophoretically onto a polyvinylidene difluoride membrane (Immobilon; Millipore, Bedford, MA, USA), the membrane was incubated with anti-fimbriae rabbit serum solution (1:1000 dilution), and then incubated with HRP-conjugated goat anti-rabbit IgG Ab (SouthernBiotech, Birmingham, AL, USA). TMB substrate solution (Nakalai Tesque Inc., Kyoto, Japan) was used to reveal the positive bands. An affinity mini-column Cellufine ET clean S and L (JNC corporation, Tokyo, Japan) was used to remove endotoxin from the purified *r*FimA solution, and a Limulus Color KY Test Wako (Fujifilm Wako Pure Chemical Corporation, Osaka, Japan) gave a reading of less than 0.1 endotoxin units of LPS per 1 μg of plasmid or *r*FimA.

### Nasal adjuvant

The plasmid pUNO1-mFlt3LGa (pFL) consists of the pUNO1 vector plus the full-length murine FL cDNA gene (InvivoGen, San Diego, CA, USA). The plasmid was purified using the EndoFree Plasmid Giga kit (QIAGEN, Valencia, CA, USA) [20, 30]. A synthetic ODN containing CpG motif 1826 (CpG ODN) (FASMAC Co., Ltd., Kanagawa, Japan) was used as a nasal adjuvant.

### Nasal immunization and sample collection

Eight- (young adult) and 48- (aging) week-old mice were nasally immunized with PBS containing 5 μg of *r*FimA plus 50 μg of pFL and 10 μg of CpG ODN as mucosal adjuvant (double adjuvant, DA) three times at weekly intervals. Control mice were immunized nasally with 5 μg of *r*FimA alone. All mice were immunized under intraperitoneal anesthesia of hydrochloric acid medetomidine (0.3 mg/kg), midazolam (4 mg/kg) and butorphanol tartrate (5 mg/kg). Plasma and saliva samples were collected 7 days after the last nasal immunization. Saliva was obtained from mice following i.p. injection of 100 μg of sterile pilocarpine/PBS solution as described previously [20, 30].

### Detection assays for *r*FimA-specific IgA ab responses

To assess *r*FimA-specific Ab levels, saliva and plasma samples were collected 7 days after the last immunization and were then subjected to ELISA as described previously [20, 30, 31]. Briefly, 96-well microtest assay plates (BD Biosciences, Oxnard, CA, USA) were coated with 1 μg/ml of *r*FImA in PBS. After incubating serial dilutions of samples, horseradish peroxidase-conjugated goat anti-mouse IgA or IgG Ab (Southern Biotechnology Associates Inc., Birmingham, AL, USA) was added to the wells. The color reaction was developed using 2,2′-azino-bis(3-ethylbenzothiazoline-6-sulphonic acid) (ABTS) substrate buffer for 15 min at room temperature. Endpoint titers were expressed as the reciprocal log_2_ of the last dilution that gave an OD_415_ of 0.1 greater than background. Mice were euthanized by cervical spine fracture dislocation under inhaled isoflurane anesthesia, and mononuclear cells were isolated from sublingual glands (SLGs), periglandular lymph nodes (PGLNs) and submandibular glands (SMGs) 1 week after the final immunization, and were then subjected to an enzyme-linked immunospot (ELISPOT) assay to enumerate the numbers of *r*FimA-specific IgA Ab-forming cells (AFCs) [20, 30–32]. In brief, mononuclear cells from SMGs and SLGs were isolated by combination of an enzymatic dissociation procedure with collagenase type IV (0.5 mg/ml; Merck KGaA, Darmstadt, Germany) followed by discontinuous Percoll® (Amersham Biosciences, Arlington Heights, IL, USA) gradient centrifugation [17, 30–32]. PGLNs were removed aseptically and the cells were then isolated using a mechanical dissociation method using gentle teasing through stainless steel screens as described previously [20, 32]. Ninety-six-well nitrocellulose plates (Millititer HA; Millipore, Bedford, MA, USA) were coated with 1 μg/ml solution of *r*FImA for analysis of anti-FimA-specific IgA AFCs.

### Flow cytometric analysis

In order to determine the numbers of CD11c^+^ DCs, aliquots of mononuclear cells (2–10 X 10^5^ cells) isolated from NALT, SLGs, SMG and PGLNs were stained with FITC-conjugated anti-mouse CD11c monoclonal Ab (BD Biosciences, San Jose, CA, USA). These samples were then subjected to flow cytometric analysis (FACSVerse®; BD Biosciences) [20, 22, 30, 31]. Typical FACS plots and gating strategy are provided in Additional file 1: Figure S1.

### Blocking assay for binding of live *P. gingivalis* 381 cells to salivary statherin

Statherin composing of 43 amino acids was artificially synthesized according to peptide sequence (FASMAC Co. Ltd., Kanagawa, Japan). Enriched saliva samples were prepared in order to determine the functionality of FimA-specific SIgA Abs, SIgA Ab. Saliva from 8- and 48-week-old non-immunized mice or mice immunized nasally with *r*FImA plus DA or *r*FimA alone were applied to a protein G affinity mini-column (Protein G HP SpinTrap; GE Healthcare Bio-Sciences AB) and saliva run through the column was used as the SIgA-enriched saliva samples. One hundred microliters of statherin in PBS (100 μg/mL) was coated to an opaque 96-well plate, and the plate was blocked with 1% BSA in PBS after washing with PBS three times. One hundred microliters of SIgA Ab enriched saliva samples (protein concentration; 1.0 mg/mL) or whole saliva was respectively mixed with 100 μL of live *P. gingivalis* cells (0.5 × 10^8^ cells) and incubated at room temperature for 1 h. The respective mixtures were subsequently added to the statherin-coated 96-well plate, and the plate was incubated for 1 h at room temperature. After each well of the plate was washed with PBS three times, the number of live *P. gingivalis* cells bound to statherin of each well was assessed based on quantitation of adenosine triphosphate (ATP) from live *P. gingivalis* cells by luminescence measurement of luciferase using a Bac Titer-Glo Microbial cell viability assay (Promega Co., Madison, WI, USA). The number of 5 × 10^7^ live cells is equivalent to 350 relative light units (RLU) of luminescence. The binding activity was calculated by subtracting the value of non-specific binding of saliva samples from 8- or 48-week-old non-immunized mice.

### Statistical analysis

The data are expressed as the mean ± standard error of the mean (SEM). All mouse groups were compared to control mice with an unpaired Mann-Whitney U test using GraphPad Prism version 7 (GraphPad software, San Diego, CA, USA). *p* values of < 0.05 were considered statistically significant.

## Results

### Induction of *r*FimA-specific SIgA ab responses in NALT and salivary gland-associated tissues of young adult and aging mice administered *r*FimA plus DA

**Fig. 2:**
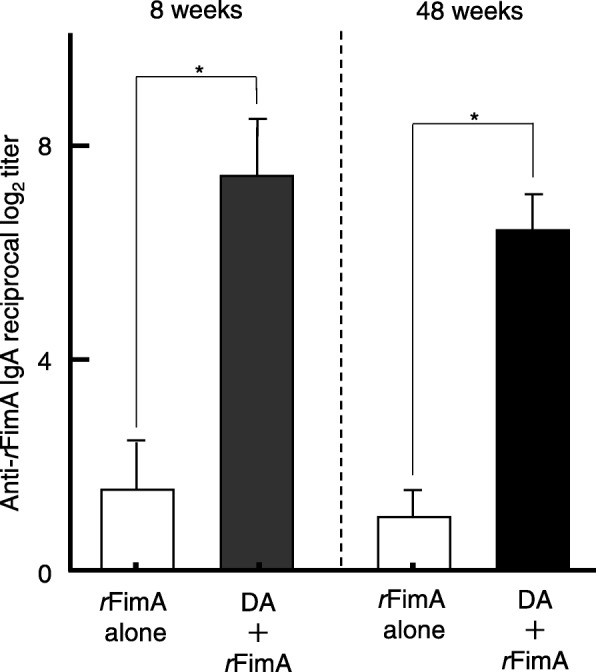
*r*FimA-specific salivary IgA Ab responses in young adult and aging mice. BALB/c (8- and 48-week-old) mice were nasally immunized three times at weekly intervals with *r*FimA (10 μg) plus pFL (50 μg) and CpG ODN (10 μg) (closed column), or *r*FimA (10 μg) alone (open column). Seven days after the last immunization, the levels of *r*FimA-specific IgA Abs in saliva were determined by *r*FimA-specific ELISA. The values shown are the mean ± SE (*n* = 20). *p < 0.05, when compared with mice group given *r*FimA alone

**Fig. 3:**
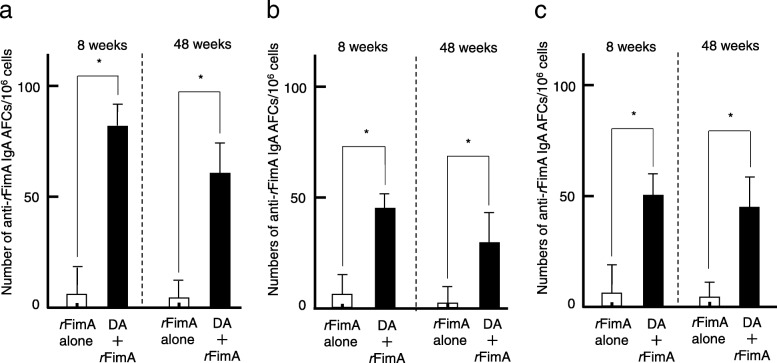
Enumeration of *r*FimA-specific IgA Ab forming cells (AFCs) in mucosal lymphoid tissues. Numbers of *r*FimA-specific IgA AFCs in SLGs (**a**), SMGs (**b**) and PGLNs (**c**) of 8- and 48-week-old mice immunized nasally with *r*FImA plus pFL and CpG ODN or *r*FimA alone. The values shown are the mean ± SE (*n* = 20). *p < 0.05, when compared with mice group given *r*FimA alone

**Fig. 4:**
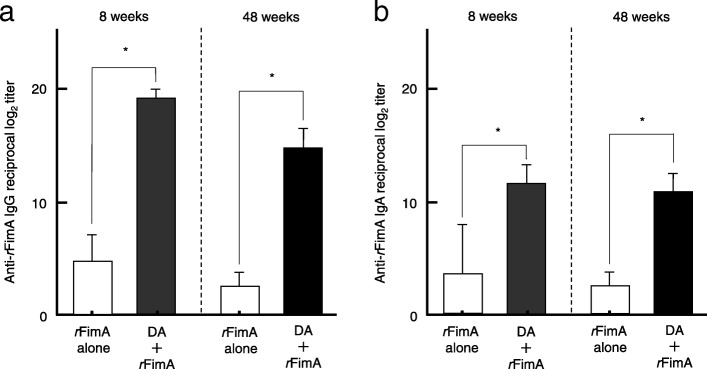
*r*FimA-specific IgG and IgA Ab responses in young adult and aging mice. BALB/c (8- and 48-week-old) mice were nasally immunized as described in Fig. 2 legend. Seven days after the last immunization, the levels of *r*FimA-specific plasma IgG (**a**) and IgA (**b**) Abs were determined by *r*FimA-specific ELISA. The values shown are the mean ± SE (*n* = 20). *p < 0.05, when compared with mice group given *r*FimA alone

We initially examined whether nasal administration of DA would enhance *r*FimA-specific SIgA Ab responses in 8- and 48-week-old mice. Nasal immunization with 5 μg of *r*FimA plus a combination of pFL and CpG ODN resulted in significantly increased levels of salivary *r*FimA-specific SIgA Ab responses in 8-week-old mice compared to those in identically aged mice given nasal *r*FimA alone (Fig. 2). Of interest, elevated levels of salivary *r*FimA-specific SIgA Ab responses were also observed in 48-week-old mice nasally immunized with *r*FimA plus DA, comparable to those responses seen in 8-week-old mice (Fig. 2). In contrast, *r*FimA-specific IgG Abs were not detected in saliva of 8- or 48-week-old mice immunized nasally with *r*FimA plus a combination of pFL and CpG ODN (data not shown). Upregulation of salivary *r*FimA-specific IgA Ab responses were further supported at the single cell level. Thus, elevated numbers of *r*FimA-specific IgA AFCs were detected in SLGs (Fig. 3a), SMGs (Fig. 3b) and PGLNs (Fig. 3c) of 8- and 48-week-old mice administered nasal *r*FimA plus DA. These results clearly show that the DA system effectively elicits *r*FimA-specific SIgA Ab responses in the nasal mucosa and salivary gland-associated tissues in young adult and aging mice. Notably, higher numbers of *r*FimA-specific IgA AFCs were detected in SLGs of 8- and 48-week-old mice (Fig. 3a) compared to those in SMGs (Fig. 3b). Since nasal immunization is known to induce systemic immunity in addition to the mucosal immune responses, *r*FimA-specific Ab responses in plasma and spleen were examined. Nasal *r*FimA plus DA elicited significantly increased levels of *r*FimA-specific IgG (Fig. 4a) and IgA Ab (Fig. 4b) responses in plasma and spleen (data not shown) of 8- and 48-week-old immunized mice compared to the responses of 8- and 48-week-old mice administered nasal *r*FimA alone. Taken together, our results show that the DA system effectively induces *r*FimA-specific mucosal IgA and systemic IgG and IgA Ab responses.

### Increases of CD11c^+^ DCs in NALT and salivary gland-associated tissues with nasal *r*FimA plus DA

Since our previous studies showed that a combination of pFL and CpG ODN as nasal adjuvants preferentially expands CD11c^+^ DCs in mucosal inductive and effector tissues [22, 30], we next examined the frequency of CD11c^+^ DCs in the various mucosal tissues of 8- and 48-week-old mice administered *r*FimA plus DA. As shown in Table 1, nasal immunization of *r*FimA plus DA significantly increased the frequency of CD11c^+^ DCs in NALT, SLGs and PGLNs of 8- and 48-week-old mice administered *r*FimA plus DA compared to identically aged mice given *r*FimA alone. Interestingly, the dramatic expansion of DC populations was observed in SLGs and PGLNs as well as NALT. Taken together, these data indicate that nasal administration of *r*FimA plus DA preferentially expands the numbers of CD11c^+^ DCs in mucosal inductive and effective sites of young adult and aging mice.

**Table 1:** Comparison of the proportion of CD11c^+^ DCs in various mucosal lymphoid tissues

		% of CD11c^+^ DCs			
Mice	DA	NALT	SLGs	SMGs	PGLNs
8 weeks	+	6.7 ± 1.1 *	11.2 ± 2.5 *	2.7 ± 1.3	14.1 ± 3.9 *
	–	1.9 ± 0.3	4.1 ± 1.4	1.9 ± 0.6	2.5 ± 0.3
48 weeks	+	5.9 ± 1.0 *	9.7 ± 2.1 *	2.5 ± 1.0	12.2 ± 4.1 *
	–	1.2 ± 0.4	2.8 ± 0.9	1.4 ± 0.5	1.9 ± 0.3

The proportions of CD11c^+^ DCs in mucosal inductive tissues (NALT), salivary glands (SLGs, SMGs) and periglandular lymph nodes (PGLNs) of mice given nasal *r*FimA plus a combination of pFL and CpG ODN (*n* = 20) or *r*FimA alone (*n* = 20) were examined. Young (8 weeks) and aging (48 weeks) mice were nasally immunized weekly for three consecutive weeks with 50 μg *r*FimA, 50 μg pFL, and 10 μg CpG ODN, respectively. One week after the final immunization, mononuclear cells from NALT, SLGs, SMGs and PGLNs were stained with FITC-anti-CD11c mAbs and subjected to flow cytometry analysis by FACSVerse®. *p < 0.05 when compared with immunized mice with *r*FimA alone

### Inhibition of live *P. gingivalis* 381 binding to salivary statherin by *r*FImA-specific salivary SIgA abs

**Fig. 5:**
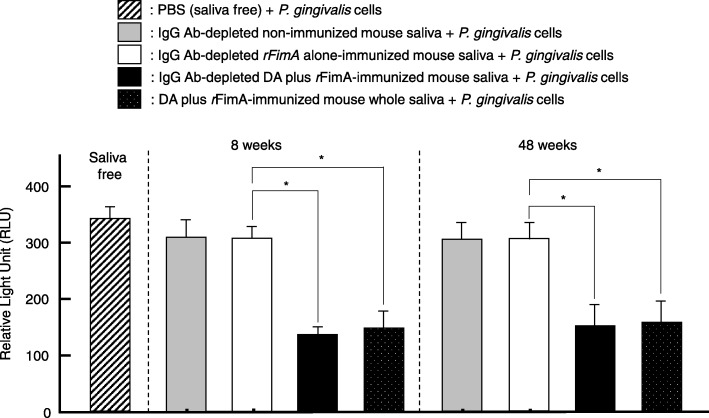
Blocking assay for live *P. gingivalis* cells binding to statherin. The inhibition of live *P. gingivalis* cell-binding to statherin by IgA-enriched saliva of 8- or 48-week-old mice nasally immunized with *r*FimA plus DA or *r*FimA alone was examined. ATP amounts of live *P. gingivalis* cells were determined by luminescence using the Bac Titer-Glo Microbial cell viability assay. The numbers of 5 × 10^7^ live cells are equivalent to 350 relative right unit (RLU) of the luminescence. The values shown are the mean ± SE (*n* = 5). *p < 0.05, when compared with mice group given *r*FimA alone

To examine the functionality of *r*FimA-specific salivary SIgA Abs, a live *P. gingivalis* cells binding assay was performed using a statherin-coated 96-well plate. SIgA Ab enriched saliva samples from 8-week-old mice administered nasal *r*FimA plus a combination of pFL and CpG ODN showed significantly reduced levels of ATP compared to those from 8-week-old mice given nasal *r*FimA alone (Fig. 5). Further, enriched IgA Ab saliva samples from 48-week-old mice nasally immunized with *r*FimA plus DA showed reduced ATP activity, indicating a significant reduction in the number of live *P. gingivalis* cells bound to the statherin-coated plate (Fig. 5). In addition, whole saliva from 8- and 48-week-old mice administered nasal *r*FimA plus DA showed essentially the same levels of ATP activities compared to those of SIgA enriched saliva (Fig. 5). These results show that *r*FimA-specific salivary IgG Abs do not play a key role in preventing *P. gingivalis*-binding to statherin. These results show that salivary *r*FimA-specific IgA Abs induced by nasal immunization with *r*FimA plus DA effectively blocked live *P. gingivalis* cell binding to a salivary protein.

## Discussion

Aging is a known risk factor of periodontal diseases [33] as well as various systemic diseases including atherosclerotic cardiovascular disease [34] and chronic obstructive pulmonary disease [35]. Indeed, declining immune function in mammals with increasing age affects the prevalence and severity of periodontal disease. In this study, we examined whether nasal administration of *r*FimA plus a combination of pFL and CpG ODN (double adjuvant, DA) as mucosal adjuvants could induce SIgA Abs to FimA of *P. gingivalis* in saliva of young adult and aging mice. Further, we investigated the functionality of salivary SIgA Abs induced by nasal *r*FimA plus DA and live *P. gingivalis* cells*.*

Flt3 ligand (FL) is a hematopoietic growth factor that has emerged as a potential immunomodulator [36–38]. Moreover, FL has been reported to increase DC populations and enhance the Ag-presenting activity of DCs [39, 40]. We have previously shown that nasal delivery of a plasmid expressing FL cDNA (pFL) plus pneumococcal surface protein (PspA) elicited increased numbers of DCs and PspA-specific SIgA Ab responses in the naso-oral cavity of young adult mice [41]. In addition, this study demonstrated that PspA-specific SIgA Abs induced by pFL as mucosal adjuvant are essential to preventing nasal carriage of *Streptococcus pneumoniae* [41]. Of importance, when CpG ODN, known as a TLR9 ligand, was added to pFL, this double adjuvant (DA) system potentiated mucosal and systemic immune responses to OVA [22], PspA [23, 30, 42] and influenza hemagglutinin Ag [43], mediated by the expansion and activation of CD11^+^ DCs in aged mice. Based on these findings [22, 23, 30, 41, 43], we hypothesized that nasal immunization with *r*FimA plus DA could induce Ag-specific functional SIgA Ab responses in the oral cavity of young adult and aging mice. Our results showed remarkably enhanced levels of *r*FimA-specific SIgA Ab induction in saliva of aging and young adult mice administered nasal *r*FimA plus DA compared to age-matched mice given nasal *r*FimA alone (Fig. 2). In addition, significantly higher numbers of *r*FimA-specific IgA AFCs were seen in SLGs of mice given nasal DA (Fig. 3a) compared to another mucosal effector tissue, namely SMGs (Fig. 3b). Based upon these finding, SLGs should be considered a mucosal effector tissue in addition to SMGs, which are known as one of the major mucosal effector tissues for the production of IgA Abs in the oral cavity [32]. Thus, our study is the first to demonstrate that nasal delivery of DA induces Ag-specific IgA AFCs in SLGs, which contributes to protective immunity in the oral cavity.

As expected, nasal administration of *r*FimA plus DA elicited a higher frequency of CD11c^+^ DCs in NALT, PGLNs, SLG and SMGs of young adult and aging mice compared to mice administered *r*FimA alone. Among them, both NALT and PGLNs contained significantly increased numbers of DCs compared to SLGs and SMGs. These results indicate that DCs activated by nasal *r*FimA plus DA play an important role as Ag-presenting cells in the mucosal inductive tissues (ie., NALT and their draining lymph nodes, i.e., PGLNs) for the activation of CD4^+^ T cells. Thus, these effector CD4^+^ T cells could migrate into the mucosal effector tissues (e.g., SLGs and SMGs) to activate IgA^+^ B cells for their differentiation into plasma cells. We are currently investigating Th1- and Th2-type cytokine production by CD4^+^ T cells in both mucosal inductive and effector tissues for their contribution to the induction of Ag-specific IgA Ab responses, as previous studies reported that the DA system exhibited a balanced Th1- and Th2-type cytokine response in both young adult and aged mice [22, 30].

Since we previously showed that *P. gingivalis* FimA protein binds specifically to a salivary statherin-coated plate [13], we next examined the interactions between live *P. gingivalis* 381 cells and *r*FImA-specific salivary SIgA Abs elicited by nasal *r*FimA plus DA in young adult and aging mice. Preincubation of *P. gingivalis* cells with enriched SIgA Abs from saliva of mice administered nasal *r*FimA plus DA resulted in reduced binding to staterin-coated plates (Fig. 5). These results suggest that *r*FImA-specific salivary SIgA Abs induced by nasal DA as mucosal adjuvant are functional and potentially inhibit the adherence of *P. gingivalis* cells to the tooth surface of young adult and aging mice.

## Conclusion

In summary, the current study clearly shows that nasal vaccination with *r*FimA plus DA elicits DC-mediated *r*FimA-specific SIgA Ab responses in saliva of young adult and aging mice. Importantly, we shed further light on *r*FimA-specific IgA Abs for providing effector function to prevent binding of *P. gingivalis* cells to the salivary protein statherin. These findings suggest that the nasal pFL and CpG ODN system can be an effective nasal adjuvant strategy to enhance salivary Ag-specific immunity in adolescents and older individuals. Furthermore, nasal vaccination with *r*FimA plus pFL and CpG ODN has potential utility in regulating *P. gingivalis-*mediated inflammatory responses in the oral cavity.

## Additional file

Additional file 1: **Figure. S1.** The typical FACS plot and gating strategy in SLGs and NALT. In FACS analysis, mononuclear cells from SLGs and NALT were gated to lymphocytes by using the forward- and side-scatter properties, and were subsequently analyzed for the populations of CD11c^+^ cells. (PPTX 4047 kb)

## Data Availability

All data generated or analyzed during this study are included in this article.

## Abbreviations

Ab: Antibody
Ag: Antigen
CpG ODN: CpG oligodeoxynucleotide 1826
DCs: Dendritic cells
FL: Flt3 ligand
*P gingivalis*: *Porphyromonas gingivalis*
*r*FimA: recombinant FimA
SIgA: Secretory-IgA
