# Supplementary material for: Nasal double DNA adjuvant induces salivary FimA-specific secretory IgA antibodies in young and aging mice and blocks Porphyromonas gingivalis binding to a salivary protein
Source: BMC Oral Health. 2019 Aug 19;19:188. doi: 10.1186/s12903-019-0886-2 (PMC6700810; doi:10.1186/s12903-019-0886-2)
Supplement: Supplementary file 1 — Figure. S1. The typical FACS plot and gating strategy in SLGs and NALT. In FACS analysis, mononuclear cells from SLGs and NALT were gated to lymphocytes by using the forward- and side-scatter properties, and were subsequently analyzed for the populations of CD11c+ cells. (PPTX 4047 kb) [file 12903_2019_886_MOESM1_ESM.pptx]

## Slide 1
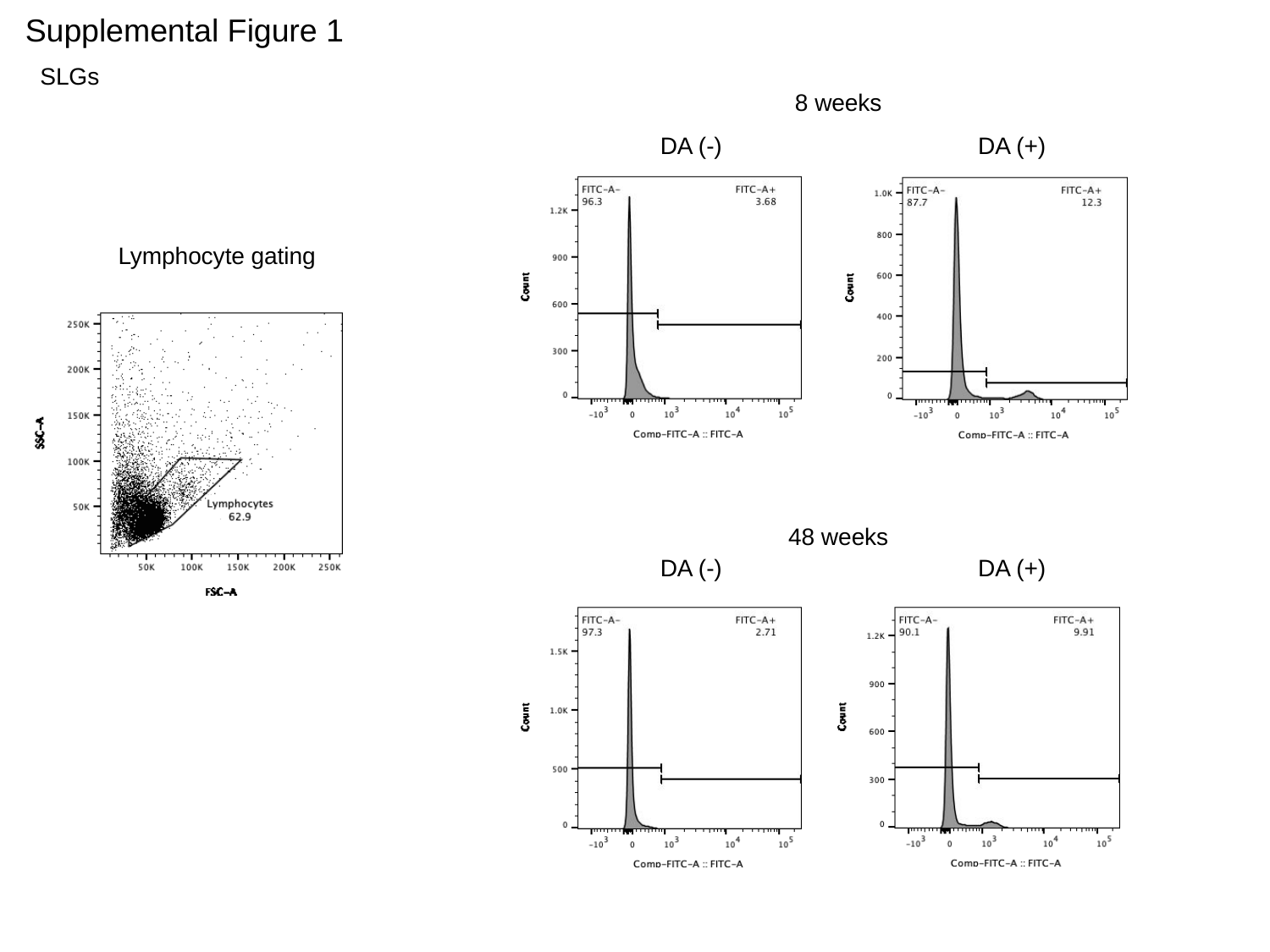

Supplemental Figure 1
SLGs
8 weeks
DA (-)
DA (+)
Lymphocyte gating
48 weeks
DA (-)
DA (+)

## Slide 2
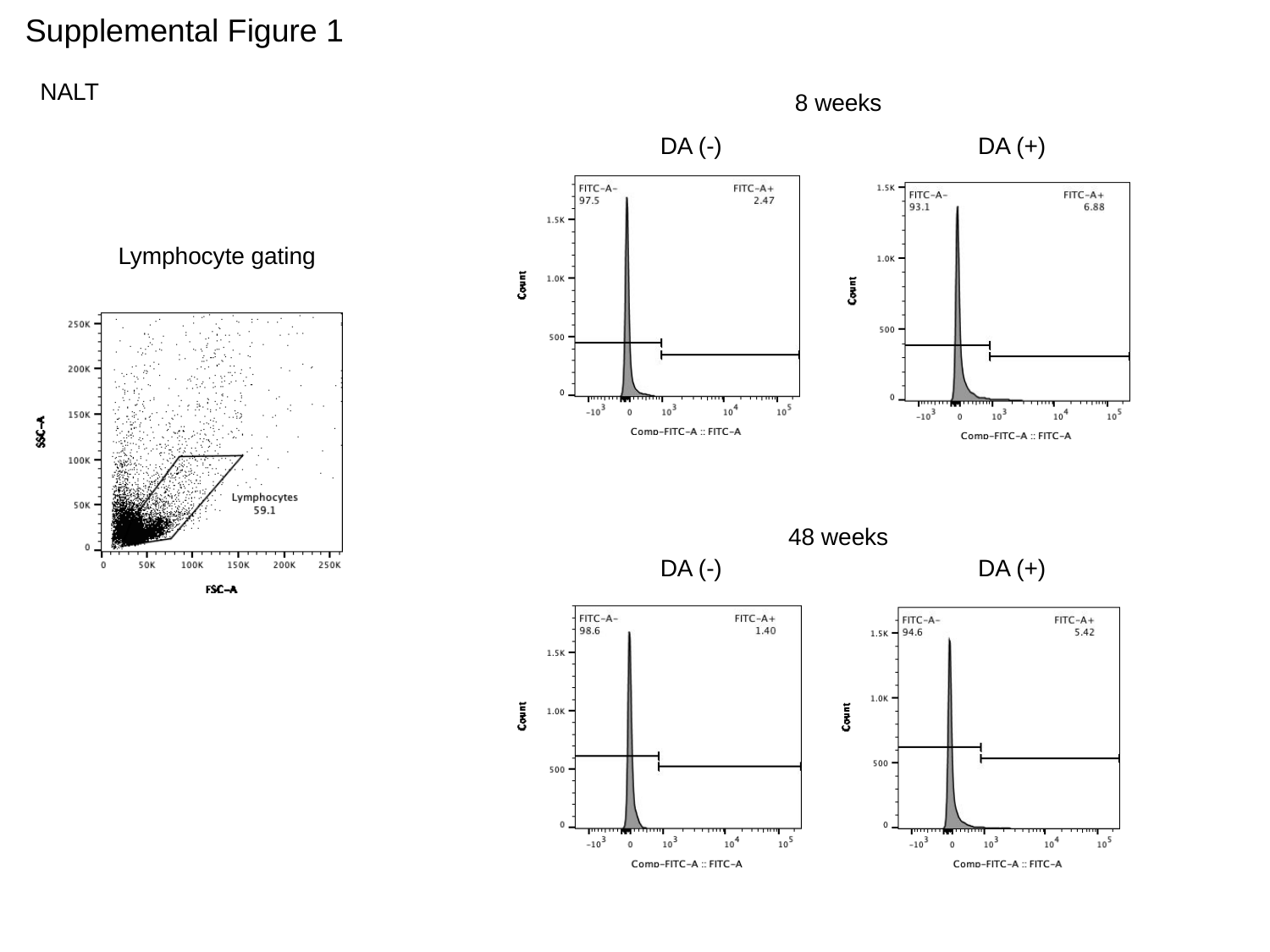

Supplemental Figure 1
NALT
8 weeks
DA (-)
DA (+)
Lymphocyte gating
48 weeks
DA (-)
DA (+)
